# Endoscopic ultrasound-guided fine-needle biopsy as a tool for studying the intra-tumoral microbiome in pancreatic ductal adenocarcinoma: a pilot study

**DOI:** 10.1038/s41598-021-04095-w

**Published:** 2022-01-07

**Authors:** Chia-Sheng Chu, Chi-Ying Yang, Chun-Chieh Yeh, Ro-Ting Lin, Chi-Ching Chen, Li-Yuan Bai, Mien-Chie Hung, Chun-Che Lin, Chun-Ying Wu, Jaw-Town Lin

**Affiliations:** 1 Digestive Medicine Center, China Medical University Hospital, 2 Yuh-Der Road, Taichung, 404 Taiwan; 2 Department of Surgery, China Medical University Hospital, Taichung, Taiwan; 3 College of Public Health, China Medical University, Taichung, Taiwan; 4 Division of Hematology and Oncology, Department of Internal Medicine, China Medical University Hospital, Taichung, Taiwan; 5 School of Medicine, College of Medicine, China Medical University, Taichung, Taiwan; 6 The University of Texas MD Anderson Cancer Center, Houston, TX USA; 7 Institute of Biomedical Informatics, National Yang Ming Chiao Tung University, Taipei, Taiwan

**Keywords:** Pancreatic cancer, Clinical microbiology

## Abstract

A new approach by investigating the intra-tumoral microbiome raised great interest because they may influence the host immune response and natural history of the disease. However, previous studies on the intra-tumoral microbiome of pancreatic ductal adenocarcinoma (PDAC) were mostly based on examining the formalin-fixed paraffin-embedded tumor specimens. This study aims to investigate the feasibility of using endoscopic ultrasound-guided fine-needle biopsy (EUS-FNB) as a complementary procedure of surgical biopsy to obtain adequate fresh pancreatic cancer tissue for intra-tumoral microbial research. This was a prospective pilot study performed at a single tertiary referral center. We obtained pancreatic cancer tissue by EUS-FNB and surgical biopsy, respectively. We amplified the V3-V4 hyper-variable region of bacterial 16S ribosomal ribonucleic acid (rRNA) genes, constructed a pair-end library, and performed high-throughput sequencing. From August 2020 to November 2020, nine eligible patients with PDAC were enrolled in this study. The intra-tumoral microbiome profile was successfully generated from the PDAC cancer tissue obtained by EUS-FNB as well as by surgical biopsy. There was no significant difference in intra-tumoral alpha-diversity or bacterial taxonomic composition between tissues obtained by EUS-FNB and by surgical biopsy. EUS-FNB can collect sufficient fresh cancer tissue for microbiome analyses without complication. The intra-tumoral microbiome profile in tissues obtained by EUS-FNB had similar alpha-diversity and taxonomic profiles with those obtained by surgical biopsy. It implicated, except for surgical biopsy, EUS-FNB can be another valid and valuable tool for studying intra-tumoral microbiome in patients with resectable and unresectable PDAC.

## Introduction

In recent years, there was a remarkable increase in the number of studies investigating the gut microbiome and cancer. It ranged from oncogenesis, cancer progression, outcome prediction to resistance to anticancer therapies^1–3^. Moreover, cancer patients seem to harbor a specific microbiome composition in the tumor niche which differs from healthy controls^4–7^. Nejman et al. found that intra-tumoral microbiome composition is diverse and cancer type-specific^4^. Riquelme et al. disclosed the intra-tumoral microbiome composition of pancreatic ductal adenocarcinoma (PDAC) patients. They identified a specific intra-tumoral microbiome signature predicting the long-term survivorship of PDAC^6^. However, studies on the intra-tumoral microbiome of PDAC were mostly based on examining the formalin-fixed paraffin-embedded (FFPE) tumor specimens obtained from patients who underwent surgical resection^4,6^. The risk of contamination with the environmental microbiota can hardly be avoided when handling the FFPE tumor specimens in a retrospective way. Ensuring that fresh tumor tissues are obtained in a sterile way is the basis of microbial research of PDAC.

PDAC is usually diagnosed late or detected until with metastases, and only a small proportion of PDAC patients can receive curative surgery. Therefore, most PDAC patients cannot provide fresh and sufficient cancer tissue for microbial studies. Endoscopic ultrasound-guided tissue acquisition become the irreplaceable tool in the diagnostic algorithm of solid pancreatic lesions. Endoscopic ultrasound-guided fine-needle aspiration (EUS-FNA) and endoscopic ultrasound-guided fine-needle biopsy (EUS-FNB) have the same safety profile, but EUS-FNB bring the better diagnostic accuracy than EUS-FNA^8^. Recently, EUS-FNB has assumed a growing role in the diagnosis and management of PDAC^9,10^. It can usually provide sufficient materials for cytological and histological examination of cancerous tissue in patients with unresectable PDAC^11–13^.

Whether EUS-FNB can be used as a complementary procedure to obtain adequate fresh pancreatic cancer tissue to investigate the intra-tumoral microbiome remained unclear. Therefore, we conducted a prospective study to investigate the intra-tumoral microbiome profile of pancreatic cancerous tissue obtained by EUS-FNB and surgical biopsy. We compared the yield rates, adverse events, and complications between these two procedures.

## Materials and methods

This was a prospective pilot study performed at a single tertiary referral center (China Medical University Hospital, Taichung, Taiwan). All EUS-FNB procedures and surgical operations were performed at China Medical University Hospital in accordance with the guideline of European Society of Gastrointestinal Endoscopy and operative regulations, respectively. The patient considered eligible for this study included patients with suspected pancreatic cancer by computed tomography or magnetic resonance imaging. Patients were excluded if they were unable to provide informed consent or were using antibiotics or probiotics before the procedure. Written informed consent was obtained from each patient or family. This study was approved by the Institutional Review Boards, China Medical University Hospital. (CMUH109-REC3-026).

### Human tumor specimens

During the surgical operation, we performed an ultrasound-guided core-needle biopsy for the pancreatic cancerous tissue with a 14-gauge needle. All surgical specimens were sterilely immersed in the lysis buffer containing 2.5% tris-HCI, 2.0% EDTA, 0.5% sodium dodecyl sulfate, and 95% distilled H_2_O, and were immediately sent for microbial analysis.

EUS-FNB was performed by an experienced endoscopist using a linear array echoendoscope (Olympus GF-UCT260, Olympus Medical Systems, Tokyo, Japan) while the patient was conscious sedated. The pancreatic lesion was carefully examined to assure no major vessels within the needle pathway before puncture in color Doppler mode. A 22-gauge needle (Acquire, Boston Scientific Corporation, Natick, Massachusetts, United States) was used for tissue sampling. The pancreatic lesion was identified and then punctured under EUS guidance. When the needle was inserted into the lesion, the stylet was slowly withdrawn. The FNB specimens were collected without negative pressure after 20 to 40 back-and-forth movements by fanning technique. In the first pass, the tissue specimens were immediately immersed in the lysis buffer, and it was delivered immediately for microbial analysis. In the second and third passes, the tissue specimens were collected and fixed with 10% neutral buffered formalin for histological examination.

### DNA extraction, bacterial 16S rRNA sequencing and microbiome analysis

Tumor samples were kept on ice and transferred to a laboratory for DNA extraction in accordance with manufacturer’s protocol, which was done by using Real Genomics DNA Extraction Kit YGE100R (RBC Bioscience Corp., New Taipei City, Taiwan). The isolated DNA aliquot was stored at − 80 °C before 16 s ribosomal ribonucleic acid (rRNA) gene sequencing. DNA concentration and quality were evaluated by NanoDrop ND-1000 (Thermo Scientific, Wilmington, DE, USA). The hypervariable region V3-V4 of bacterial 16S rRNA genes was amplified by polymerase chain reaction using bar-coded universal primers 341F (F, forward primer; 5-CCTACgggNggCWgCAg-3′) and 805R (R, reverse primer; 5′-gACTACHCgggTATCTAATCC-3′). Library construction and sequencing of amplicon DNA samples were committed to Germark Biotechnology (Taichung, Taiwan). A pair-end (2 × 300) library (insert size of 465 base pairs for each sample) was constructed with TruSeq Nano DNA Library Prep kit (Illumina, San Diego, CA, USA), and high-throughput sequencing was performed on an Illumina MiSeq 2000 sequencer with MiSeq Reagent Kit v3 (Illumina). The bioinformatics analysis of 16S rRNA amplicon was conducted by Germark Biotechnology (Taichung, Taiwan). Briefly, on a per-sample basis, paired-end reads were merged using USEARCH (v8.0.1623)^14^, with a minimum overlap of read pair set at 8 base pairs (bp). Merged reads were quality-filtered with Mothur (v1.34.1)^15^ to remove reads shorter than 400 bp or longer than 550 bp, as well as reads with a minimum average quality score lower than 27. In addition, reads containing an ambiguous base or homopolymer exceeding 8 bp were excluded. Chimera detection was performed using USEARCH (reference mode and 3% minimum divergence) and removed from further analysis. Quality-filtered and non-chimeric reads were analyzed (UPARSE pipeline)^16^ to generate operational taxonomic units (OTUs) per sample (at 97% identity level). The OTU representative sequences were searched against the Greengenes 13_5 database by using USEARCH global alignment to identify the corresponding taxonomy of the best hit. OTUs without a hit or with only a weak hit, that is, the function “(% sequence identity + % alignment coverage)/2” less than 93^17^, was excluded from the following analysis. Diversity indices (e.g., Shannon, Simpson, Inv Simpson) were estimated with the R package phylosea^18^.

### Statistical analyses

The alpha-diversity in the tissues obtained by EUS-FNB and surgical biopsy was compared using the Mann-Whitney test. Hierarchical clustering (via complete-linkage algorithm) of microbiomes was conducted using the Bray-Curtis distance of OTU-level relative abundance profile, based on which principal coordinates analysis (PCoA) was also performed using the R package ade4^19^. A p-value less than 0.05 was considered statistically significant.

## Results

From August 2020 to November 2020, nine eligible patients with PDAC were enrolled in this study. They were six men and three women, with a mean age of 61.8 (47–76) years. Five patients had PDAC located in the head of pancreas, one in the body, and three in the tail. Five PDAC patients were diagnosed with stage III and four with stage IV. We used EUS-FNB to obtain pancreatic cancer tissues from six patients. We obtained tissue samples from four patients during surgery. One patient underwent both EUS-FNB and surgery. This patient underwent EUS-FNB, and the result revealed atypical glands initially; the patient was later confirmed as adenocarcinoma by a subsequent surgical operation. Five patients (5/6, 83.3%) who underwent EUS-FNB were confirmed PDAC, while all four patients (4/4, 100%) in the surgical group were diagnosed as PDAC. There was no internal bleeding, pancreatitis, and other adverse events after EUS-FNB. No internal bleeding nor other complications occurred in subjects who underwent surgery.

**Figure 1:**
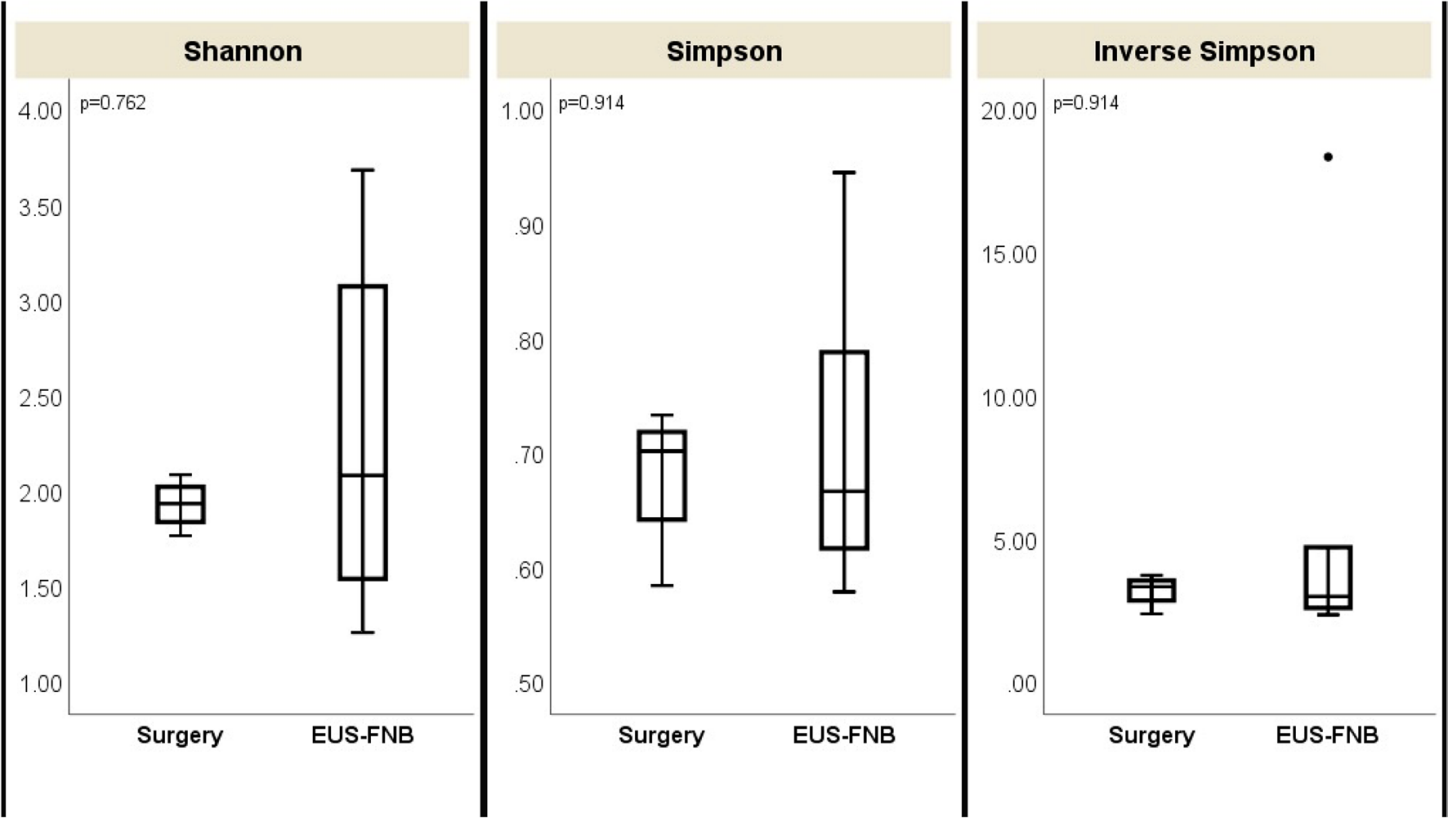
There was no significant difference in alpha-diversity (represented by Shannon, Simpson, and inverse Simpson indices) between endoscopic ultrasound-guided fine-needle biopsy (EUS-FNB) group and surgical group.

**Figure 2:**
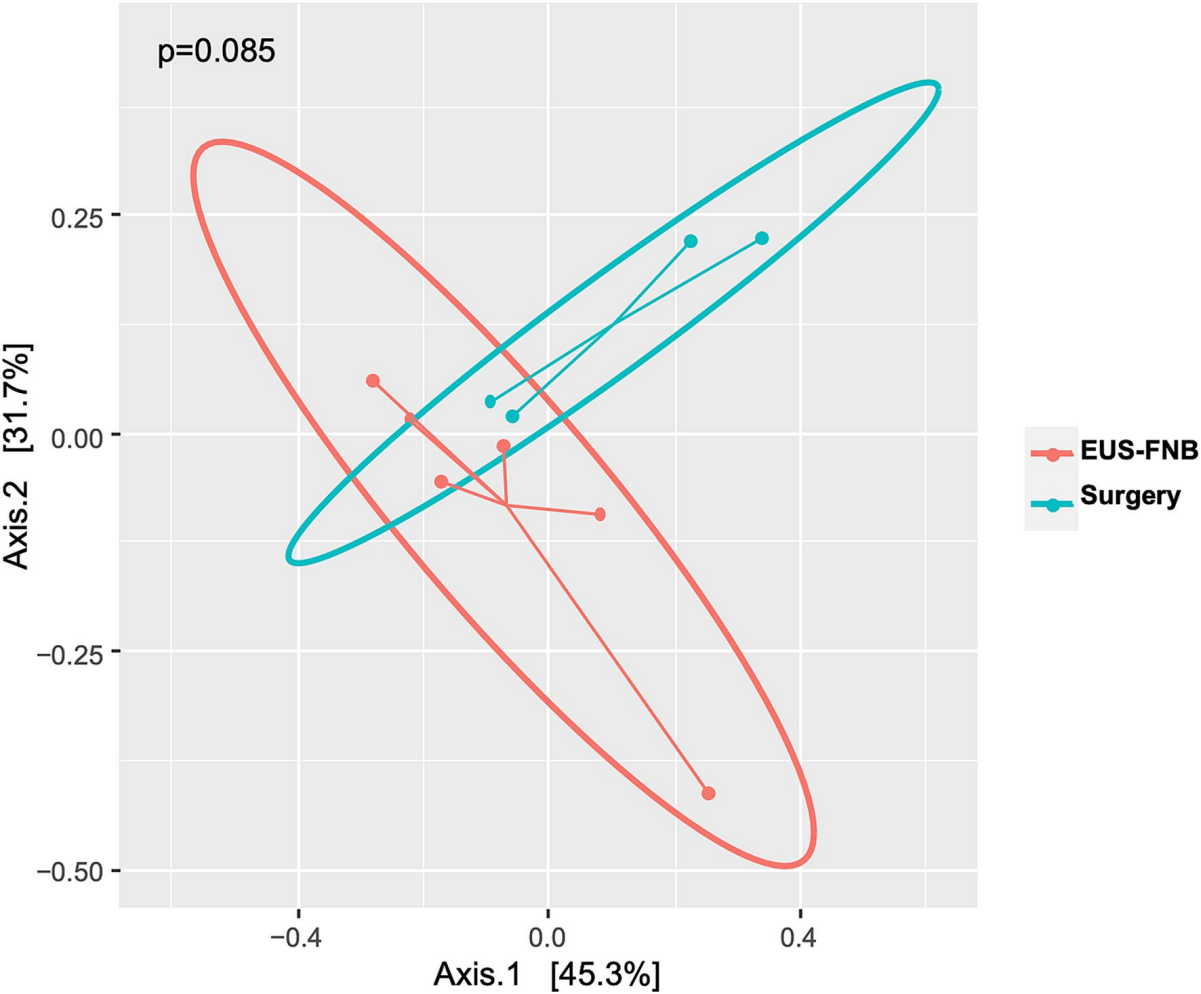
Principal coordinates analysis (PCoA) revealed no significant difference (P = 0.085) in bacterial operational taxonomic unit (OTU) composition at genus level between endoscopic ultrasound-guided fine-needle biopsy (EUS-FNB) group and surgical group.

The intra-tumoral microbiome profile was successfully generated from the PDAC cancer tissue obtained by EUS-FNB as well as by surgical biopsy. For microbial profiling, a total of 1.2 million pair-end reads were generated, of which 962 thousand reads passed quality filtering and were not chimera. To determine microbial diversity and composition, reads were aligned to the Greengenes database, and non-bacterial sequences were removed. The number of observed OTUs and the intra-tumoral bacterial diversity (alpha-diversity, represented by Shannon, Simpson, and inverse Simpson indices) were not significantly different between the two groups (Fig. 1), indicating the intra-tumoral bacterial abundances were similar between EUS-FNB and surgical groups. PCoA also revealed no significant difference in bacterial OTU composition between EUS-FNB and surgical biopsy (P = 0.085) (Fig. 2). In other words, the intra-tumoral bacterial composition between these two groups were close.

## Discussions

The associations of gut microbiota with various human diseases and various types of cancers have been widely investigated, especially their roles in tumorigenesis and cancer progression. The microbiome has emerged as a new potential biomarker for cancer diagnosis, risk stratification, and prognosis. Previous studies usually investigated the association between the gut microbiome and PDAC by collecting the fecal^3,20–22^ or salivary^23–25^ samples to determine the microbial profile. Some bacteria, such as *Porphyromonas*, *Fusobacterium*, *Aggregatibacter*, *Prevotella*, and *Capnocytophaga*, were found to play a role in the development of PDAC^26^. However, the microbiome profiles obtained from saliva and feces were inconsistent and conflicting, probably due to various technique of sample extraction, processing, and data analysis^27^.

Recently, a new approach by investigating the intra-tumoral microbiome raise great interest. Geller et al. showed the presence of *Gammaproteobacteria* in PDAC might be responsible for the tumor resistance to gemcitabine^5^. The gut microbiome also plays a significant role in antitumor immune responses and predicts the efficacy of immune-checkpoint inhibitors in cancer patients^28,29^. Riquelme et al. identified an intra-tumoral microbiome signature (*Pseudoxanthomonas*–*Streptomyces*–*Saccharopolyspora*–*Bacillus clausii*) which was predictive of long-term survivor in PDAC. They demonstrated that PDAC microbiome composition, which cross-talked to the gut microbiome, could influence the host immune response and natural history of the disease^6^. Chakladar et al. outlined the intra-tumoral microbiome of 187 PDAC samples through large-scale sequencing data from The Cancer Genome Atlas (TCGA), and they found potentially cancer-promoting or immune-inhibiting microbes—most of them belonged to *Proteobacteria* phylum^30^. Nejman et al. reported that intra-tumoral microbiome composition is diverse and cancer type-specific. They analyzed the intra-tumoral microbiome of 1,526 samples from seven human tumor types, including breast, lung, ovary, pancreas, melanoma, bone, and brain tumors. Bacteria belonging to the *Firmicutes* and *Bacteroidetes* phyla were the most abundant species in colorectal tumors, while *Proteobacteria* dominated the microbiome of PDAC^4^.

However, previous studies on the intra-tumoral microbiome of PDAC were mostly based on the FFPE tumor specimens obtained during surgical resection^4,6^. The majority of PDAC patients were diagnosed at advanced stages and thus precluded surgical resection. Masi et al. compared the results of microbiome profile in FFPE specimens obtained by surgical biopsy and EUS-FNB using Decontam^31^ (http://github.com/benjjneb/decontam), an open-source R package, to remove contaminant DNA sequences. They found there was no significant difference in alpha-diversity, beta-diversity, or taxonomic profiles between EUS-FNB and surgical biopsy in three patients with matched samples^32^. They claimed that EUS-FNB could substitute surgical biopsy in the PDAC tissue sampling for microbial research. This prospective study confirmed that both two methods can collect sufficient cancer tissue for microbiome analyses.

Since the EUS-FNB has the better diagnostic accuracy than EUS-FNA^8^, and the EUS-FNB with the newest generation of needles has better histological procurement yield than older ones while performing solid pancreatic lesion biopsy^33,34^. In our study, we collected fresh PDAC tissue via EUS-FNB with newest generation of EUS-FNB needles and surgical biopsy. Both EUS-FNB and surgical biopsy can collect sufficient fresh cancer tissue for microbiome analyses without major complication. Furthermore, the intra-tumoral microbiome profile from EUS-FNB had similar alpha-diversity and taxonomic profiles with surgical biopsy. There are some limitations of our study including the small number of patients and the possible rare bias from 16S rRNA amplicon sequencing. Thus, further larger studies with whole genome sequencing are warranted.

In conclusion, except for surgical biopsy, EUS-FNB can be another valid and valuable tool for studying intra-tumoral microbiome in patients with resectable and unresectable PDAC.

## Data Availability

No additional data are available.

## Abbreviations

PDAC: Pancreatic ductal adenocarcinoma
FFPE: Formalin-fixed paraffin-embedded
EUS-FNA: Endoscopic ultrasound-guided fine-needle aspiration
EUS-FNB: Endoscopic ultrasound-guided fine-needle biopsy
rRNA: Ribosomal ribonucleic acid
bp: Base pairs
OTU: Operational taxonomic unit
PCoA: Principal coordinates analysis
